# Safety and preliminary results of perioperative chemotherapy and hyperthermic intraperitoneal chemotherapy (HIPEC) for high-risk gastric cancer patients

**DOI:** 10.1186/1477-7819-10-195

**Published:** 2012-09-19

**Authors:** Wilson L Costa, Felipe J F Coimbra, Héber S C Ribeiro, Alessandro L Diniz, André Luís de Godoy, MariaDirleiFS Begnami, Milton J B Silva, Marcelo F Fanelli, Celso A L Mello

**Affiliations:** 1 Department of Abdominal Surgery, Hospital A. C. Camargo, Rua Antonio Prudente, 211 Liberdade CEP, 01501-900, Sao Paulo, Brazil; 2 Department of Surgical Pathology, Hospital A. C. Camargo, Rua Antonio Prudente, 211 Liberdade CEP, 01501-900, Sao Paulo, Brazil; 3 Department of Clinical Oncology, Hospital A. C. Camargo, Rua Antonio Prudente, 211 Liberdade CEP, 01501-900, Sao Paulo, Brazil; 4 Department of Abdominal Surgery, Hospital A. C. Camargo, Rua Antonio Prudente, 211 Liberdade CEP, 01501-900, Sao Paulo, Brazil

**Keywords:** Gastric cancer, HIPEC, Perioperative chemotherapy

## Abstract

**Background:**

Gastric cancer relapse occurs in about 30% of the patients treated with gastrectomy and D2-lymphadenectomy, mainly as distant or peritoneal metastases. Hyperthermic intraperitoneal chemotherapy (HIPEC) has been associated with an improvement in survival and lower peritoneal recurrence, albeit with increased morbidity. The aim of this study is to report the preliminary results of the association of perioperative chemotherapy, radical surgery and HIPEC in high-risk gastric patients in a single institution.

**Methods:**

Treatment protocol was started in 2007 and included patients younger than 65 years old, with good performance status and gastric adenocarcinoma with serosa involvement and lymph node metastases, located in the body or antrum. Patients should receive three preoperative cycles of DCF (Docetaxel 75 mg/m^2^, Cisplatin 75 mg/m^2^ and continuous intravenous infusion of 5-Fluorouracil 750 mg/m^2^ for 5 days), followed by gastric resection with D2-lymphadenectomy, hyperthermic intraperitoneal chemotherapy with Mytomicin C 34 mg/m^2^ and three more postoperative cycles of DCF.

**Results:**

Ten patients were included between 2007 and 2011. Their median age was 47 years old and six were male. Nine were staged with cT4 cN + tumors and one as cT3 cN+. Nine patients completed all three preoperative chemotherapy cycles. Eight individuals were treated with a total gastrectomy and the other two had a distal gastrectomy, all having HIPEC. Postoperative morbidity was 50%, with no deaths. Regarding postoperative chemotherapy, only 5 patients completed three cycles. With a median follow-up of 25 months, three relapses were identified and 7 patients remain disease-free, two with more than 4 years of follow-up.

**Conclusion:**

The association of perioperative systemic and intraperitoneal chemotherapy plus radical surgery is a feasible multimodality treatment, with acceptable morbidity. With a longer follow-up and a larger group of patients, we hope to be able to determine if it also influences survival outcomes and patterns of recurrence.

**Mini-Abstract:**

The association of perioperative chemotherapy, gastric resection and D2-lymphadenectomy and hyperthermic intraperitoneal chemotherapy proved to be associated with acceptable morbidity. For survival analysis, a longer follow-up is needed.

## Background

The surgical treatment of gastric cancer in western countries has been associated with 5-year survival rates of between 20 and 30% [1]. This number is quite small compared with that observed in Eastern historical series, of around 60% [2], and in recent randomized studies of stage II and III patients who had 3-year survival of over 70% [3]. This difference has been attributed to a higher incidence of proximal tumors and more advanced disease at diagnosis [1]. However, the extent of surgery seems to have a major role in it. In Japan, for patients with tumors in stages IB through IIIC, according to the seventh edition of the so-called tumor, node, metastasis (TNM) staging system [4], the guidelines for surgical treatment recommend resection with D2-lymphadenectomy [5]. On comparison of Japanese survival rates with those in Western cancer centers where extended lymphadenectomy has been routinely performed, the numbers are very similar [6].

Even with optimal surgery, more than 30% of patients will have tumor relapse in the first two years of follow-up, primarily as peritoneal or distant visceral metastasis [7,8]. These results favor a multimodal approach in the treatment of gastric cancer. The first relevant Western trial was the INT 0116, which demonstrated an improvement in overall and disease-free survival with adjuvant chemoradiation after a negative-margin resection [9]. Surgical quality control was a weak point in this trial, in which only 10% of patients underwent a D2 dissection. Interestingly, this group of individuals had no survival benefit with adjuvant treatment [9]. In 2006, the Medical Research Council adjuvant gastric infusional chemotherapy (MAGIC) trial showed that patients who had perioperative chemotherapy and surgery had better overall and disease-free survival [10]. Some of the benefits of neo-adjuvant treatment were revealed in this study, as it allows early treatment of micrometastases and increases the probability of an R0 resection [11].

Another modality in the multidisciplinary treatment of gastric cancer is intraperitoneal chemotherapy. It has been shown to improve survival in patients treated with D2-lymphadenectomy and to reduce peritoneal recurrence [12]. A recent meta-analysis associated hyperthermic intraperitoneal chemotherapy, with or without early postoperative intraperitoneal chemotherapy, and superior overall survival numbers, albeit with increased morbidity [13].

The aim of this study is to report the initial experience of a single Brazilian Cancer Center with a protocol of multimodal treatment in selected patients with locally advanced gastric cancer. It includes the association of perioperative chemotherapy, gastrectomy with D2-lymphadenectomy and hyperthermic intraperitoneal chemotherapy (HIPEC).

## Methods

### Patients

Only patients under 65 years of age with Karnofsky performance status (KPS) over 70% were included in this protocol. Laboratory results included normal Hb/Ht and leucogram and preserved renal (creatinine ≤ 1.5 mg/dl, BUN ≤ 30 mg/dl) and hepatic (bilirubin < 2 mg/dl) functions. They also had to have microscopically confirmed diffuse-type adenocarcinoma, located in the body or antrum of the stomach and have been clinically staged as non-metastatic T3 N + or T4 tumors, according to the TNM staging proposed by the American Joint Committee on Cancer [4].

Exclusion criteria included prior history of any neoplasm; impairment from clinical conditions; tumors of the gastric stump; gastroesophageal junction tumors; T1-2-3 N0 or T1-2 N1 lesions, and M1 disease at diagnosis.

Pretreatment staging included upper endoscopy with biopsy, chest and abdominal multi-slice computer tomography (CT), laparoscopy and endoscopic ultrasound with fine needle aspiration, if necessary. The laparoscopic procedure should have included the placement of a 10-mm infra-umbilical port and one or two additional 5 to 12 mm ports, as necessary. A thorough examination of peritoneal and liver surfaces should have been performed followed by peritoneal washing for cytology analysis. Enlarged regional lymph nodes were reported, but not biopsied.

Beginning in 2007, ten patients were included in this protocol. All of them were staged with upper gastrointestinal endoscopy, thoracic and abdominal CT. A laparoscopy was performed in nine patients; one patient refused to have the laparoscopic procedure prior to neoadjuvant therapy. Eight patients had endoscopic ultrasound examination. Nine patients were staged as cT4 cN + and one as cT3 cN + .

Written informed consent was obtained for all patients included in this study. The Ethics Committee at A. C. Camargo Hospital, São Paulo, Brazil, approved the protocol.

### Treatment

Patients who fit all eligibility criteria were seen by a group of oncologists and started on preoperative chemotherapy comprising docetaxel, cisplatin and 5-fluorouracil (DCF) as follows: docetaxel 75 mg/m^2^, cisplatin 75 mg/m^2^ and continuous intravenous infusion of 5-fluorouracil 750 mg/m^2^ for 5 days. The treatment plan included three preoperative cycles of chemotherapy followed by surgery and three postoperative cycles of chemotherapy. Treatment-related hematological toxicity was evaluated according to CTCAE v4.0.

Re-staging took place prior to surgery and it included upper endoscopy, thoracic and abdominal CT and endoscopic ultrasound, when available. Partial response was defined as decreased gastric wall thickening, lymph node size or number on CT, or lesion measure of more than 20% on upper endoscopy, or as significant relief from clinical symptoms. Gastric wall involvement, and number and size of perigastric lymph nodes were also analyzed by endoscopic ultrasound.

Surgical treatment consisted of total or distal gastrectomy and D2-lymphadenectomy. Frozen sections of the negative margins were obtained. Extended resections, such as splenectomy or pancreatectomy, were performed only when deemed necessary so that negative margins could be achieved.

After resection and reconstruction, just prior to temporary abdominal closure, a perfusion system was prepared and HIPEC was administered. Our HIPEC system is a closed circuit, allowing perfusate circulation with a variable dynamic flow of 500 to 3000 mL/minute and hyperthermic capability ranging between 38 to 45°C. A five-branched inflow catheter was inserted in the abdomen toward the subdiaphragmatic surfaces, the pericolic spaces and the pelvic recess. Conversely, a three-branched outflow catheter was directed to the diaphragmatic surfaces within the pouch of Douglas. Approximately 3 to 4 L of dialysis solution containing 34 mg/m^2^ mitomycin C was circulated for 90 minutes. Mitomycin C was used as it had been shown to have an acceptable toxicity profile on intraperitoneal use for gastric cancer [12] and it would be associated with systemic chemotherapy, which at first could increase postoperative morbidity and mortality. Temperature control was established with simultaneous positioning of three intra-abdominal thermometers, following the distribution of inflow and outflow catheters. Intra-abdominal temperature should reach numbers between 40 and 42°C in all three thermometers. Close attention was performed to the cardiac and respiratory function and an intra-esophageal thermometer was inserted to control body temperature.

Postoperative morbidity was described using the modified Clavien classification for surgical complications [14].

### Pathological analysis

All the resection specimens were evaluated according to a standardized protocol. They were opened and the macroscopically identifiable tumor or area of the stomach with scarring indicating the site of the previous tumor (tumor bed) was cross-sectioned serially at 0.5 cm intervals. These tissue sections were stained with hematoxylin and eosin and period acid-Schiff (PAS) stain was performed in some cases. Surgical resection margins, portions of the corpus and antrum, and lymph nodes were evaluated separately. The extent of the tumor (TNM classification) and the completeness of resection (R) were determined according tp the guidelines of the International Union Against Cancer (UICC).

### Follow-up

Every patient included in this study started follow-up after the last cycle of postoperative chemotherapy or for those who did not receive adjuvant therapy, after the surgical procedure. Appointments were scheduled every three months for the first two years, at a six-month interval for the next three, and yearly thereafter. Patients had to have had a physical examination, a complete blood count, liver-function testing, and abdominal and thoracic CT.

### Statistical analysis

A preliminary survival analysis was performed. Overall survival was determined and its interval was established by the time in months between surgery and death or the last hospital visit. The overall survival curves were obtained through the Kaplan-Meier method.

## Results

### Patients

Between April 2007 and December 2011, 10 patients were included and treated according to our protocol. Six of them were male and four female. Mean age was 47.6 years, range 34 to 63 years. In the beginning of treatment, the KPS was over 80% for all patients. One person had high blood pressure. Familial history was an interesting element in this cohort of patients, as there was one prior case of esophageal squamous cell carcinoma, one of laryngeal cancer, two of non-Hodgkins’ lymphoma and one of breast ductal carcinoma.

### Staging and treatment

Regarding neoadjuvant treatment, nine patients completed three cycles as proposed. One patient had his chemotherapy interrupted after two cycles, due to gastrointestinal toxicity. Table 1 describes the toxicity associated with the proposed treatment.

**Table 1:** Toxicity profile of patients treated with perioperative and hyperthermic intraperitoneal chemotherapy

**Event**	**Grade 3/4**					
	**Preoperative chemotherapy**			**Postoperative chemotherapy**		
	**1**	**2**	**3**	**1**	**2**	**3**
Adverse events, n/total	5/10	3/10	1/10	2/10	2/10	6/10
Grade 3/4 events	4/10	3/10	1/10	2/10	0/10	2/10
Non-hematological toxicity	3	3	1	2	0	1
Fatigue	0	0	0	0	0	0
Anorexia	0	1	0	0	0	0
Gastrointestinal	1	2	1	2	0	1
Mucositis	2	0	0	0	0	0
Rash	0	0	0	0	0	0
Alopecia	0	0	0	0	0	0
Fever	0	0	0	0	0	0
Hematological toxicity	1	0	0	0	0	1
Pancytopenia	0	0	0	0	0	0
Neutropenia	1	0	0	0	0	1
Total events	4	3	1	2	0	2

Results are presented as number of patients. Hematological toxicity was evaluated according to CTCAE v4.0. All grade 3/4 adverse events are described in Table 1.

Evaluation work-up after neoadjuvant chemotherapy included upper gastrointestinal endoscopy, and thoracic and abdominal CT for all patients, and endoscopic ultrasound for four of them. In seven patients a partial clinical response was observed and there were three other patients with stable disease. No disease progression was described.

Surgical treatment consisted of a total gastrectomy in eight patients and a distal resection in two. In all patients, D2-lymphadenectomy was performed with a median of 33 dissected nodes. An R0 resection was achieved in every case, and in three patients a distal pancreatectomy with splenectomy was performed. Median operative time was 450 minutes, and intraoperative blood transfusion was needed in only patient.

The overall postoperative morbidity rate was 50%. By stratifying complications as proposed by Dindo *et al*. [14], we identified three grade II, one grade IIIa and one grade IIIb events. Three patients had fever between the 7^th^ and 9^th^ postoperative days, with no identification of an infection site, were treated with empiric antibiotics, and discharged between the 10^th^ and 13^th^ day, without any new event. One patient was diagnosed with a pancreatic fistulae through the identification of an abnormal discharge in the closed suction drain placed during surgery, and confirmed by a fluid amylase examination. No additional treatment was necessary. Another patient, the second in our series, had an intra-abdominal abscess that required re-laparotomy. There was no postoperative mortality.

The treatment protocol established that all patients received three more cycles of DCF. However, the toxicity profile in the postoperative setting was much more severe. Only five patients completed three more cycles as proposed. One patient refused adjuvant chemotherapy, two had their treatment interrupted after the first cycle due to gastrointestinal toxicity, and the other two patients received only cisplatin and 5-fluorouracil during the second and third cycle.

### Treatment response and preliminary survival results

Pathological analysis demonstrated one pathologic complete response and three patients with ypT1-2 tumors, but in the others over 50% of viable tumor (minor response) was identified in the surgical specimen.

Median follow-up was 25 months, ranging from 9 to 60 months. One patient had tumor relapse after 4 months of treatment, with distant lymph node and brain metastasis, and died two months later. Two patients developed peritoneal relapse, one 90 days after surgery, and the other after 15 months of follow-up. Seven patients are alive and without disease at the time of writing this report (Table 2).

**Table 2:** Clinical and pathological data of treated patients

**Patient number**	**Age (years)**	**Gender**	**TNM staging**	**Re-staging**	**Morbidity**	**Pathology staging**	**Follow-up (months)**	**Status**
1	56	F	T4 N+	SD	Postoperative fever	T2 N0 M0	28	Alive, without disease
2	62	M	T4 N+	PR	Intraabdominal abscess	T4a N2 M0	60	Alive, without disease
3	34	F	T4 N+	PR		T2 N0 M0	10	Death due to cancer
4	47	M	T4 N+	SD	Pancreatic leakage	T4a N3a M0	26	Death due to cancer
5	49	F	T4 N+	PR		T4a N3a M0	33	Alive, without disease
6	50	M	T4 N+	PR	Postoperative fever	T1N1M0	52	Alive, without disease
7	39	M	T4 N+	SD		T4a N3b M0	9	Death due to cancer
8	49	M	T4 N+	PR		T0 N0 M0	22	Alive, without disease
9	56	M	T4 N+	PR	Postoperative fever	T4aN3aM0	20	Alive, without disease
10	34	F	T3 N+	PR		T3N3aM0	24	Alive, without disease

F, female; M, male.

Pathology staging: T, tumor; N, node; M, metastasis.

## Discussion

Our study demonstrated the preliminary results of a multimodality treatment protocol that involved the association of perioperative systemic and intraoperative hyperthermic chemotherapy with radical surgical treatment. Optimal surgery for gastric cancer includes a gastric resection with adequate margins [15] and D2-lymphadenectomy, which has been associated with higher survival rates in the East [16], and also in some Western countries [17]. This is the standard for curative surgery at our institution, also with the emphasis on obtaining a lower ratio between the metastatic and dissected lymph nodes (N-ratio), as demonstrated in a previous paper [18]. Even with adequate surgical treatment, gastric cancer still recurs in more than 30% of patients [7,8]. However, patterns of disease recurrence tend to differ with the extent of lymphadenectomy. In a large retrospective series with 2328 patients treated with D2 dissection, overall recurrence was 28%. Peritoneal relapse was the most common (43.9%), followed by haematogenous and loco-regional events, which happened in 34.3% and 32.5% of the patients [7]. In one large Western series, among 1172 patients treated with an R0 resection for gastric cancer, there was a 42.3% recurrence. Loco-regional recurrence was the most common, in 54% of the cases, followed by distant (51%) and peritoneal (29%) relapse. However, this series included a high number of patients with esophagogastric junction tumors (44.4%) and 19% of the individuals had a D1-lymphadenectomy [8]. In other studies, when a D2-lymphadenectomy was performed in all patients, peritoneal and distant recurrences were more common; nonetheless, when D1-dissections were included, loco-regional relapses tended to increase [7,8,19].

Neoadjuvant and adjuvant therapies are associated with different patterns of disease relapse. The addition of postoperative chemoradiation in the INT0116 trial was related to superior overall and disease-free survival and a significant decrease in local recurrence, but not in peritoneal or distant metastasis. Moreover, adjuvant chemoradiation somehow managed to compensate a poor surgical treatment, since a D2-lymphadenectomy was performed in only 10% of the patients [9]. In the MAGIC trial, the group treated with perioperative chemotherapy and surgery had lower incidence of loco-regional recurrence and distant metastasis. Statistical analysis of this data was not performed [10]. This same study demonstrated some of the advantages of neoadjuvant treatment, as more than 90% of the patients completed all three preoperative cycles of chemotherapy, but only 42% completed all six cycles. This can be explained by the nutritional impairment that gastrectomy patients tend to suffer after surgery, which may delay or even cancel any adjuvant treatment. Moreover, in a recent European trial, individuals who were treated with preoperative chemotherapy had tumor downstaging and a higher number of R0 resections and N0 tumors [20]. At last, the Eastern trial of adjuvant chemotherapy with S-1 demonstrated an improvement in 3-year overall and disease-free survival, and it was also the first study in which a systemic treatment was associated with a decrease in peritoneal recurrence [3].

HIPEC, along with surgical cytoreduction, is part of the standard treatment for peritoneal mesothelioma and pseudomyxoma peritonei, and also a very important tool in the treatment of peritoneal carcinomatosis of colorectal and ovarian cancer [21]. Recently, surgical cytoreduction and HIPEC for the treatment of peritoneal carcinomatosis of gastric cancer was associated with a 20% 5-year survival in selected patients [22].

When patients are treated with a D2-lymphadenectomy, loco-regional recurrences are less common, and peritoneal relapse may occur in up to 40% of the individuals [7]. That is the rationale for the use of intraperitoneal chemotherapy as a tool in the multimodal treatment of patients with M0 gastric cancer.

An Eastern randomized trial investigated the use of early postoperative intraperitoneal chemotherapy with mitomycin C and 5-fluorouracil in stage II and III patients after curative surgery. There was no survival benefit in the whole set of patients. However, in the subgroup of individuals with stage III disease, there was a significant improvement in overall survival (49.1% vs. 18.4%). Decreasing incidence of peritoneal dissemination after surgery with early postoperative intraperitoneal chemotherapy was also observed [23]. Another Eastern randomized trial investigated the use of HIPEC as part of the surgical procedure. It included 141 patients and identified a significant improvement in overall survival in the multimodality group after 2, 4 and 8 years (88% vs. 77%; 76% vs. 58%; 62% vs. 49%, respectively). Death due to peritoneal recurrence was significantly reduced (one patient in the multimodality group and sixteen in the surgery-only group) [12]. In an attempt to assess the effectiveness and safety of adjuvant intraperitoneal chemotherapy in the treatment of gastric cancer, a meta-analysis of 13 studies was published in 2007. There was an improvement in overall survival with the addition of HIPEC, followed or not by early postoperative intraperitoneal chemotherapy. There were also higher risks of intra-abdominal abscess and neutropenia [13].

As the results of surgical and multimodality studies have demonstrated, we believe that the set of combined treatment to be instituted in a population should focus on loco-regional recurrence when sub-optimal surgery is performed, or on peritoneal and distant relapse, when patients are routinely treated with D2-lymphadenectomy, as is the case in our service.

Morbidity could be increased with the addition of HIPEC, which prompted us to focus on the toxicity associated with it. As expected, over 90% of the patients completed the first three cycles of chemotherapy, with only one individual having grade 3 toxicity events. The postoperative morbidity was high (50%), but only two patients had grade III complications, with one of them demanding exploratory surgery. The association of neoadjuvant and intraperitoneal chemotherapy has already been investigated in a Western phase II study, in which preoperative cisplatin and 5-fluorouracil was followed by gastrectomy with D2-lymphadenectomy and early postoperative intraperitoneal chemotherapy. There were 38 patients in the study, and around 80% of them had T3 and node-positive disease. The treatment was not associated with an increase in morbidity, but was associated with higher R0 resection rates and a 39% 5-year survival [24]. Recently, an Eastern study investigated the use of neoadjuvant S-1 followed by surgery and intraperitoneal paclitaxel in 12 patients with positive peritoneal cytology, with no postoperative deaths. In 75% of them, no peritoneal metastases were found during surgery and their peritoneal washing was negative [25].

Our results should be interpreted with caution, as the small number of patients and a relatively short time of follow-up may limit any conclusion regarding survival. However, we believe that this study demonstrates that the association of perioperative systemic and intraperitoneal hyperthermic chemotherapy plus radical surgery is a feasible multimodality treatment, with acceptable morbidity. With a longer follow-up and a larger group of patients, we hope to be able to determine if it also influences survival outcomes and patterns of recurrence.

## Conclusion

The association between perioperative chemotherapy, radical surgery and intraperitoneal hyperthermic chemotherapy is a safe and feasible multimodality treatment.

## Competing interests

All the authors in this study declare no conflict of interest.

## Authors’ contributions

ES - Wilson L Costa Jr, Héber S C Ribeiro, Alessandro L Diniz, André Luís de Godoy, Milton J B Silva. FG - Felipe J F Coimbra, Maria Dirlei F S Begnami, Marcelo F Fanelli, Celso A L Mello. All authors read and approved the final manuscript.
